# Special Issue “Molecular Mechanisms and Treatment of Allergic Reactions”

**DOI:** 10.3390/ijms26157461

**Published:** 2025-08-01

**Authors:** Georgina Hopkins, Lucy C. Fairclough

**Affiliations:** School of Life Sciences, University of Nottingham, Nottingham NG7 2RD, UK; georgina.hopkins@nottingham.ac.uk

Allergic diseases are increasing worldwide and affect approximately 10–30% of individuals [1,2,3,4]. Therefore, there is an increasingly urgent need to understand the mechanisms underpinning allergic sensitisation and elicitation to provide a stream of new insights into potential treatments. The Special Issue “Molecular mechanisms and treatment of allergic reactions” provides a variety of original articles and reviews focusing on advances in the allergy field. Here, six articles were included, covering aspects such as epithelial barrier dysregulation [5], the role of extracellular vesicles in atopic dermatitis [6], Platelet-Activating Factor in nasal polyps [7], the allergenicity of less well-defined proteins and their use in immunotherapy or hypoallergenic products [8,9], and the interplay between diet and co-sensitisation [10]. 

The first review article in this Special Issue focused on the interplay of the epithelial barrier, immune cells, and metabolic mediators in allergic disease [5]. The epithelial barrier is the first line of defence against environmental allergens, and chronic exposure to pollutants, chemicals, pathogens, etc., can disrupt the epithelial barrier, increasing immune interactions with allergens and thus increasing the likelihood of allergy development. The review details how damaged epithelial cells can release alarmins, such as IL-33 and TSLP, which are key Th2 pathway activators. Specifically, these pro-inflammatory signals can then activate Group 2 innate lymphoid cells (ILC2s) and eosinophils. Importantly, ILC2s and eosinophils regulate allergic inflammation through lipid mediators and are associated with lipid metabolite alterations in allergy. With current technologies allowing the linking between metabolic pathways and allergic disease phenotypes, there is a great interest in research that can facilitate drug discovery. Thus, the authors propose that immune–metabolomics can help guide future diagnostics and treatments by identifying biomarkers and pathways associated with allergic phenotypes.

Another review also focused on the epithelial barrier but specifically highlighted the role of extracellular vesicles (EVs) in the barrier and how EVs can promote, and in some cases suppress, atopic dermatitis (AD) [6]. The review provides a comprehensive overview of EVs released by the bacterium *Staphylococcus aureus*, the fungus *Malassezia sympodialis*, and host mast cells and their role in skin inflammation. It is well-known that an overabundance of *S. aureus* is a key contributor to AD, but the role of *S. aureus* EVs is a more recent concept. *S. aureus* EVs were shown to promote AD pathogenesis through increased epidermal thickening and keratinocyte necrosis to disrupt the barrier [11,12]. *S. aureus* EVs also carry biologically active betalactamase (BlaZ), an enzyme capable of inactivating beta-lactam antibiotics like penicillin and cephalosporins, and were able to transfer a transient resistance against ampicillin to surrounding bacteria [13]. *Malassezia*-derived EV research was limited compared to *S. aureus,* but the review summarised *Malassezia-*derived EVs contained allergens and promoted inflammatory cytokine production, contributing to AD. Finally, EVs released by mast cells were discussed and found to promote dendritic cell maturation and lymphocyte proliferation, reduce free IgE, and also promote cytokine production. Overall, through skin barrier damage, cytokine promotion, allergen transport, and immune cell activation, EVs were shown to both promote and, in some cases, protect against atopic dermatitis. While research in this area is still in its infancy, the studies examined in this review provide encouraging insights into how EVs released from a variety of cells play a role in both contributing to and protecting against atopic dermatitis (Figure 1).

There are two studies in this Special Issue that focused on the allergenicity of less well-defined allergens. One of these studies focused on the allergenicity of the major pollen allergen, Aln g 1, from *Alnus glutinosa* [9]. The authors explain that Bet v 1 is the primary sensitiser for birch-related pollen allergies and has been extensively researched, yet other birch pollen allergens, such as Aln g 1, are less well-defined, particularly their potential use in allergen immunotherapy (AIT). The authors demonstrate that Aln g 1 caused upregulation of epithelial-cell-derived alarmins and was able to bind hydrophobic ligands, which promotes allergic sensitisation. They go on to show, for the first time, that the allergenicity of Aln g 1 can then be reduced by the substitution of two amino acid residues, Asp27 and Leu30, in its structure, which reduced its ability to bind hydrophobic ligands, as well as IgE, in the sera of allergic patients. This work offers insights into designing hypoallergenic variants of Aln g 1 to provide safer but effective AIT.

Similarly, the other study investigated the allergenicity of another less well-defined allergen, glutenin, from *Aegilops tauschii* [8]. Wheat allergy is a major food allergy, yet the allergenicity of the ancient diploid wheat progenitor (*Aegilops tauschii*), which was used to develop common wheat, is unknown. Thus, this study exposed glutenin to Balb/c mice to investigate its allergenicity and found that the ancient wheat allergen induced a robust IgE response and systemic anaphylaxis, without adjuvants. Proteomic analysis of spleen tissue highlighted increased Th2 activation, as well as identified biomarkers associated with systemic anaphylaxis. Despite previous research suggesting that ancient wheat species are less allergenic [14], this study contradicts that hypothesis and shows that glutenin from *Aegilops tauschii* was found to be intrinsically allergenic, eliciting clinical sensitisation for systemic anaphylaxis. The authors highlight that gene modifications of glutenin may be made to reduce the allergenicity of the ancient wheat species, developing hypoallergenic wheat for wheat-allergic individuals. 

Co-sensitisation of allergens is well-known to exacerbate allergic reactions [15], but the interplay between co-sensitisation and dietary factors has not yet been established. One study evaluated co-sensitisation of house dust mites and ragweed pollen, in combination with a high-fructose diet, on airway reactivity [10]. The results indicate that co-sensitisation of the two allergens in rats exacerbates allergic asthma compared to single-allergen exposures. In addition, rats on a high-fructose diet, to promote obesity and dyslipidaemia, exhibited increased airway inflammation, with elevated serum IgE and c-reactive protein (CRP), as well as inflammatory cell infiltration with airway remodelling. The authors highlight the synergistic effect of obesity on exacerbating existing allergic asthma inflammation and therefore suggest a link between diet and environmental allergens in eliciting allergic asthma, implying management of diet may help prevent and treat allergic asthma. 

The final study included in this Special Issue investigated the association of Platelet-Activating Factor (PAF) with the pathology of chronic rhinosinusitis with nasal polyps (CRSwNP) [7]. It is known that CRSwNP involves dysregulation in the synthesis of glycerophospholipid mediators, including leukotrienes, prostaglandin, thromboxane, and PAF. Previous research suggested a link between PAF metabolism and CRSwNP pathophysiology [16,17,18]. Thus, this study conducted transcriptomic analysis of genes associated with PAF metabolism, such as enzymes involved in PAF synthesis (LPCAT1, LPCAT2, LPCAT3, and LPCAT4) and PAF degradation (PAFAH1B2, PAFAH1B3, and PAFAH2), and the gene for the PAF receptor (PTAFR). A cohort of 27 patients were recruited, including those with eosinophilic chronic rhinosinusitis (ECRS) or non-eosinophilic chronic rhinosinusitis (nonECRS), those with ECRS with aspirin-exacerbated respiratory disease (AERD), and healthy controls. Firstly, they found that hierarchical-analysis-based classification for endotype categorisation was useful in elucidating the differences in CRSwNP, as the clustering grouped patients into low–moderate type 2 inflammation and severe type 2 inflammation. Subsequent analysis of gene expression in these two groups identified a significant downregulation of LPCAT2 and an upregulation of PTAFR expression in the low-to-moderate type 2 inflammation group. In contrast, in severe type 2 inflammation patients, upregulation of LPCAT1, PAFAH1B2, and PTAFR and downregulation of PAFAH2 expression were observed (Figure 2). The findings suggest that severe type 2 inflammation is associated with heightened PAF-related pathology in CRSwNP. These results provide insights into potential therapeutic targets, e.g., anti-PAF drugs, and biomarkers such as LPCAT1 for type 2 inflammation severity. 

Overall, this Special Issue provides a diverse collection of original and review articles covering different aspects of the molecular mechanisms of allergic reactions and potential treatments. The articles encompass exciting and novel findings but also highlight the requirement for further research in this area. Specifically, the role of microbial and host EVs in allergy is of growing interest, and more research is needed to understand the mechanisms behind their influence on allergic sensitisation and elicitation and to establish their use as biomarkers for allergic disease. This Special Issue also highlights developments in hypoallergenic variants of allergens, as well as the interplay between diet and allergen co-sensitisation, but these studies are in their early stages, and more translational research is needed to establish this in a clinical setting. The Special Issue also concludes that immune–metabolomic profiling is a useful tool for future research to define specific metabolic pathways that drive allergic phenotypes and also identify biomarkers for therapeutic or diagnostic tools, such as anti-PAF therapeutics for type 2 inflammation.

## Figures and Tables

**Figure 1:**
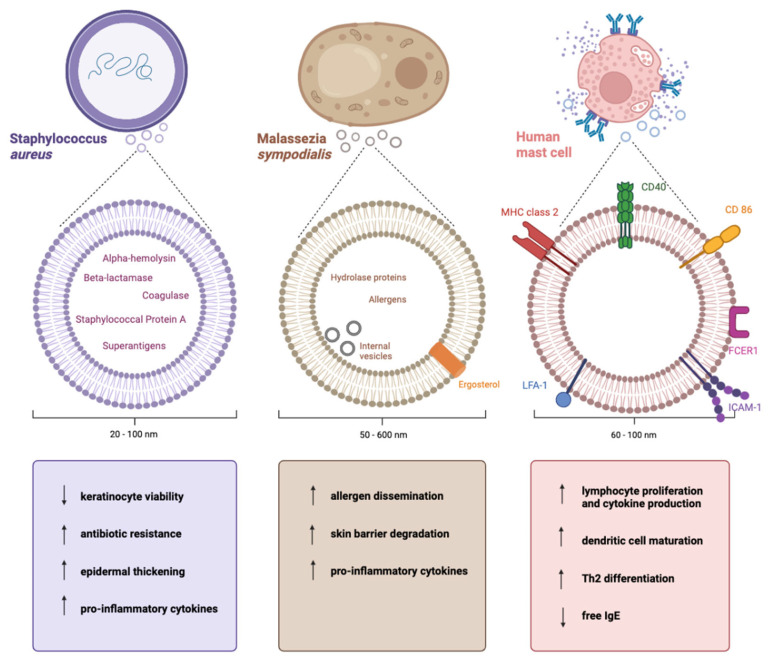
**Summary of cells of origin, characteristics, and effects of *S*. *aureus*, *M*. *sympodialis,* and mast-cell-derived EVs.** *S. aureus* EVs decrease keratinocyte viability and increase antibiotic resistance, epidermal thickening, and the release of pro-inflammatory cytokines. The direction of the effect seems to be towards the promotion of AD pathogenesis. Similarly, *Malassezia*-derived EVs increase allergen dissemination, skin barrier degradation, and the release of pro-inflammatory cytokines. However, by increasing lymphocyte activation, dendritic cell maturation, and Th2 differentiation while decreasing free IgE, host mast cell EVs seem to have a protective tendency in AD. Created using BioRender. Adopted from [6].

**Figure 2:**
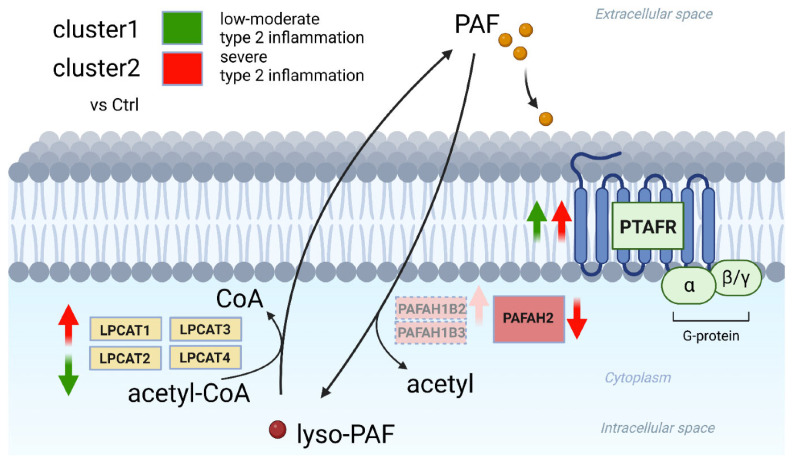
**Summary of PAF-metabolism-associated gene expression in CRSwNP**. Cluster 2 of CRSwNP, which exhibits severe type 2 inflammation, has a high PAF-associated pathophysiology with the upregulation of PAF synthesis (LPCAT1) and the downregulation of PAF degradation (PAFAH2), leading to local PAF accumulation and intensification of the effects of PAF signalling via the upregulation of PTAFR. Adopted from [7].
